# Transcriptome analysis of differences in the infection of African swine fever virus (SY-1 strain) in iPAMs and PAMs

**DOI:** 10.3389/fimmu.2025.1692373

**Published:** 2025-12-05

**Authors:** Haiying Mao, Chuxing Cheng, Yong Wang, Wenhui Zhou, Ke Zhang, Yongqi Wang, Lihao Wang, Zhenrui Song, Xiaomei Sun, Yuanfeng Zhang, Xiaotong Hu, Yumei Zhang, Zong Zou, Ya Zhao, Qiang Zhang, Meilin Jin

**Affiliations:** 1 State Key Laboratory of Agricultural Microbiology, Huazhong Agricultural University, Wuhan, China; 2 Hubei Jiangxia Laboratory, Wuhan, Hubei, China

**Keywords:** African swine fever virus, SY-1 strain, viral replication, iPAMs, PAMS, transcriptomic analysis, JPH4 and CYP1A1

## Abstract

African swine fever (ASF) is a highly contagious hemorrhagic disease caused by African swine fever virus (ASFV), which has inflicted devastating impacts on the global swine industry. Currently, no commercially available vaccines exist. A major obstacle in ASF research of mechanistic exploration and vaccine studies is the virus’s strict tropism for primary cells like porcine alveolar macrophages (PAMs), while exhibiting poor susceptibility to most immortalized cell lines. This study compared the replication and host response of the ASFV SY-1 strain in PAMs versus immortalized porcine macrophage cell lines (iPAMs) using qPCR, western blot, and early infection transcriptome data. Results revealed that the SY-1 strain replicates only minimally in iPAMs, with significantly lower replication capacity than in PAMs. Transcriptomic data demonstrated divergent host responses: iPAMs predominantly involved in in the regulation of lipid metabolism and response to oxidative stress, whereas PAMs preferentially activate cytokine signaling and immune responses. Furthermore, Functional validation indicated *JPH4* and *CYP1A1* (selected from differentially expressed genes list) as potential host factors influencing viral replication. This study delineates early host–virus dynamics in ASFV susceptible cells (PAMs) and restricted replicating cells (iPAMs), which not only provides an insight into the replication of ASFV but also improved rational design of antiviral strategies in ASF research.

## Introduction

African swine fever (ASF) is a highly virulent infectious disease caused by the African swine fever virus (ASFV) which has caused profound economic losses and operational disruptions in pig farming on a national scale (1–3). It was first identified in Kenya in 1921 (4). Following its initial emergence, ASFV spread across multiple regions, including parts of Africa and Eurasia, before reaching China. The first confirmed outbreak in China occurred in Heilongjiang Province in 2018 (5). This epidemic devastated the swine industry in China and rapidly extended to neighboring countries and territories such as South Korea, Vietnam, Mongolia, Cambodia, North Korea, Laos, and Myanmar, which considerably elevated the risk of ASF transmission throughout East Asia (6–9).

The ASFV is a large double-stranded DNA genome has 170–193 kb long (10, 11). This genomic variation may be primarily attributed to the genetic differences in the multigene families (MGFs) that exhibit extensive copy number polymorphisms across ASFV strains (12, 13). The virus encodes > 150 proteins that are categorized into four major functional groups: (1) viral structural proteins and proteins involved in viral particle formation (≥ 54); (2) enzymes and cofactors involved in genome replication and transcription; (3) proteins involved in regulation of host immunity and metabolism; and (4) MGF-encoded viral proteins (14, 15). Despite a 100-year history and extensive researcher efforts, no effective vaccines or drugs have been developed against ASFV with successful clinical outcomes, which implied that ASFV presents multiple challenges (16). Beyond its large genome and diverse proteome, a critical obstacle is the virus’ strict preference for primary cells, which inherently limits genetic manipulation or modification. This limitation explain why progress on ASFV has remained slow despite its emergence over a century ago.

In our study, we found that iPAMs exhibited lower susceptibility to SY-1 compared to PAMs. To assess the differences in viral responses between iPAMs and PAMs, we performed a comparative transcriptomic analysis during the early stages of ASFV infection and found fundamentally distinct host defense strategies. PAMs mounted a robust inflammatory response characterized by the upregulation of interleukin (*IL*) and chemokines (*CXCL*), consistent with their physiological role in initiating innate immunity. In contrast, iPAMs exhibited minimal inflammatory signaling but showed activation of autophagy-related pathways and oxidative stress. Further investigation of differentially expressed proteins showed that *JPH4*, which is a member of the endoplasmic reticulum–plasma membrane protein family (17), and *CYP1A1*, which is a member of the cytochrome P450 superfamily of enzymes (18), suggesting that these genes may be host factors in ASFV infection.

We hope to characterize the potential mechanisms of viral infection between primary and passaged macrophages by integrating multi-omics data, which provide an insight for ASFV research of replication.

## Materials and methods

### Safety protocols

All experiments involving ASFV SY-1 infection were performed in an enhanced biosafety level 3 (P3+) facility.

### Cells and virus

Porcine kidney-15 (PK15), intestinal porcine epithelial cell line-J2 (IPEC-J2), and immortalized swine pulmonary alveolar macrophages (iPAMs) were maintained in our laboratory while PAM cells were obtained from piglets weaned at 25–30 days, as previously described (19). First of all, 5 healthy weaned piglets (all experimental pigs were screened according to the standard of general grade experimental pigs to ensure that antigen-antibody tests for ASFV, PCV2, PRRSV, CSFV, and PRV were negative to exclude the potential effects of potential infections or immune disturbances on the subsequent experiments) were euthanized via Zoletil^®^50 (Virbac, French) overdose for lung collection. Secondly, the surface of the lung was wiped with 75% medical alcohol, and then filter-sterilized Phosphate-Buffered Saline (PBS) was instilled into the lungs through the trachea until the whole lung was filled with PBS and PBS was poured out of the lungs. Finally, the cells were collected by centrifugation at 1500 rpm, resuspended in medium containing 100 µg/mL streptomycin, and 100 IU/mL penicillin, and filtered through a 70µm cell strainer (Biosharp, China). The cells obtained after the above steps were primary alveolar macrophages (PAMs). The PAMs were cultured in RPMI 1640 medium (Sigma, USA) containing 10% fetal bovine serum (FBS). The PK15, iPAMs, and IPEC-J2 cells were cultured in DMEM (Sigma, USA) containing 10% FBS. ASFV SY-1 was stored in our laboratory under GenBank accession number OM161110. This study adhered to ethical guidelines and minimized animal usage through optimized protocols and the acquisition of PAMs were approved by the Research Ethics Committee, Huazhong Agricultural University, Hubei, China (202503300001).

### Detection of virus load using quantitative polymerase chain reaction

The ASFV genomic DNA copy number was determined in the cells and supernatants using qPCR by following established protocols. Briefly, PAMs and iPAMs were infected with ASFV at a multiplicity of infection (MOI) of 5 for 2 h at 37 °C. Following infection, the supernatants and cellular fractions were collected at specified time points. Viral genomic DNA was extracted using the FastPure Viral DNA/RNA Mini Kit (Vazyme Biotech, China). qPCR amplification was performed using the QuantStudio5 Real-Time PCR System (Applied Biosystems, USA). The reaction system (20 µL) contained 2 µL of FAM-BHQ1 labeled probe (5′-CCACGGGAGGAATACCAACCCAGTG-3′), 2 µL of PerfectStart II Probe qPCR Supermix UDG (TransGen Biotech, China), 5 µL of template DNA, and 11 µL of nuclease-free water.

### Binding and entry assays

To evaluate ASFV attachment, the iPAMs and PAMs (1×10^6^ cells/well) were seeded in 12-well cell culture plates and incubated with ASFV (MOI = 5) at 4 °C for 1 h to allow viral binding. The supernatants were discarded, and the cells were washed thrice with cold PBS. Viral infection was detected using qPCR. The same procedure was followed to assess viral entry. After washing the cells thrice with cold PBS, RPMI1640 medium (Sigma, USA) supplemented with 5% FBS (Gibco, USA) was added, and the cells were incubated at 37 °C for 30 min to facilitate viral entry. Finally, the cells were washed thrice with cold PBS, and qPCR was performed to determine the viral load.

### RNA sequencing

iPAMs and PAMs were seeded in a 6-well plate at a density of 3×10^6^ cells/well and infected with ASFV (MOI = 5) at 37 °C for 2 h. After incubation, the cells were washed with PBS to remove the unbound virus and maintained in fresh culture medium up to 3 or 6 hour post-infection (hpi). Total RNA was extracted using TRIzol reagent (Invitrogen, USA) according to the manufacturer’s instructions. The RNA samples were treated with DNase I to remove residual genomic DNA contamination. RNA purity and concentration were determined using a NanoDrop 2000 spectrophotometer (Thermo Scientific, USA). RNA integrity was assessed using an Agilent 2100 Bioanalyzer (Agilent Technologies, USA). Sequencing libraries were generated using the NEBNext Ultra Directional RNA Library Prep Kit (New England Biolabs, USA) by following the manufacturer’s instructions. High-quality cDNA libraries were sequenced on an Illumina HiSeq 2500 platform (Illumina, USA). Base calling was performed using CASAVA v1.8.2, and 150-bp paired-end reads were generated.

### Transcriptome analysis

Raw reads were quality-controlled by removing poly-N sequences and low-quality reads to obtain clean reads. The Q30 score and GC content of the clean reads were calculated. Then, the clean reads were mapped to the reference pig genome (Sscrofa v11.1, downloaded from Ensemble) using HISAT2 (v2.0.1) (20) with default parameters. The genome annotation file (GTF format) was included during alignment to improve transcript quantification accuracy. Differentially expressed genes (DEGs) were detected by obtaining the read counts per gene using HTSeq v0.6.1. Differential expression analysis was performed using the DESeq2 R (v1.20.0) (21) package with raw counts as input. The DEGs were identified with a corrected p-value < 0.05 and |log_6_ (fold change) | > 1. For visualization, normalized counts (e.g., FPKM) were used for downstream analyses. Gene ontology (GO) and Kyoto Encyclopedia of Genes and Genomes (KEGG) pathway enrichment analyses of DEGs were performed using ClusterProfiler with an adjusted p-value (FDR) threshold of < 0.05. The background gene set was defined as all expressed genes in the dataset.

### Small interfering RNAs and plasmids

Gene-specific siRNAs and a non-targeting control siRNA (siNC) were commercially synthesized by AuGCT DNA-SYN Biotechnology (Wuhan, China). To construct the Flag-tagged expression plasmids, cDNA sequences corresponding to the target swine genes were PCR-amplified and cloned into the pCMV-Flag vector using standard molecular cloning techniques. The sequences of all plasmid constructs were verified before experimental use. The sequences of the siRNAs and plasmids used in this study are listed in **Supplementary Table 1**.

### Reverse transcription-qPCR

Total RNA was extracted using the EasyPure Fast Cell RNA Kit (TransGen Biotech, China) according to the manufacturer’s instructions. The mRNA levels of the genes were detected using the primers listed in **Supplementary Table 2**. Reverse transcription was performed using TransScript All-in-one First-Strand cDNA Synthesis Super Mix for qPCR (RNASGen Biotech, China). RT-qPCR was performed on a QuantStudio6 system (Applied Biosystems, USA) according to the procedure recommended by the Office of International Education (OIE). GAPDH was used as an endogenous negative control, and calculations were performed using 2^-△△Ct^.

### Generation of Cas9-overexpressing iPAM cell lines

Cas9-overexpressing iPAM cell lines were established by generating lentiviral particles through the co-transfection of 293T cells with lentiviral packaging plasmids and a Cas9 expression plasmid that included the Flag-tagged plasmid. Viral supernatants harvested after 48 h were used to infect wild-type iPAMs seeded in 12-well plates. A second cycle of infection was performed after 12 h. At 24 h post-infection, the medium was replaced with complete growth medium, and the cells were cultured for an additional 36 h before selection with Blasticidin S (20 μg/mL, InvivoGen). The surviving cells were subjected to monoclonal isolation through limited dilution in 96-well plates.

### Western blotting

The cells were transfected with the plasmid, washed with cold PBS, and lysed on ice for 10 min using cell lysis buffer containing 1% protein inhibitor (MCE, USA) for western blotting and immunoprecipitation (IP) (Beyotime, China). The lysates were boiled in 5× SDS loading buffer for 10 min. The proteins were separated via 10% polyacrylamide gel electrophoresis and transferred onto polyvinylidene fluoride (PVDF) membranes, which were blocked with 2% bovine serum albumin (BSA) and incubated with an anti-Flag (1:3000) monoclonal antibody, GAPDH monoclonal antibody (1:3000), β-Actin monoclonal antibody (1:3000) (Proteintech, China) and (or) P30 polyclonal antibody (1:500) prepared by our own experiments for overnight at 4°C. Horseradish peroxidase-labeled goat anti-rabbit IgG and Horseradish peroxidase-labeled goat anti-mouse IgG (1:5000) was used as the secondary antibody.

### Thin-section electron microscopy

ASFV-infected iPAMs and PAMs were scraped off the plates and were centrifuged at 2000 rpm for 10 minutes. Cell pellets were fixed with 2.5% glutaraldehyde (in 0.1 M phosphate buffer, pH 7.4) at 4°C for at least 4 hours, followed by post-fixation with 1% osmium tetroxide for 30 minutes at room temperature. Subsequently, specimens were dehydrated through a graded ethanol series (30%, 50%, 70%, 90%, 100%). The cells were embedded in epoxy resin, and resin blocks were sectioned into 80-nm-thick ultrathin slices. Sections were mounted on copper grids and stained with 2% aqueous uranyl acetate and Reynold’s lead citrate. ASFV particles were observed and imaged using a transmission electron microscope.

### Statistical analysis

All data were derived from at least two independent experiments, with each independent experiment including three technical replicates and quantitative data are presented as mean ± standard deviation (SD). One-way and Two-way analysis of variance (ANOVA) of GraphPad Prism 8.0 was used for statistical analyses. Statistical significance was defined as P < 0.05.

## Results

### iPAMs are a relatively susceptible for SY-1 and have gene editing capabilities porcine macrophage cell line

**Figure 1:**
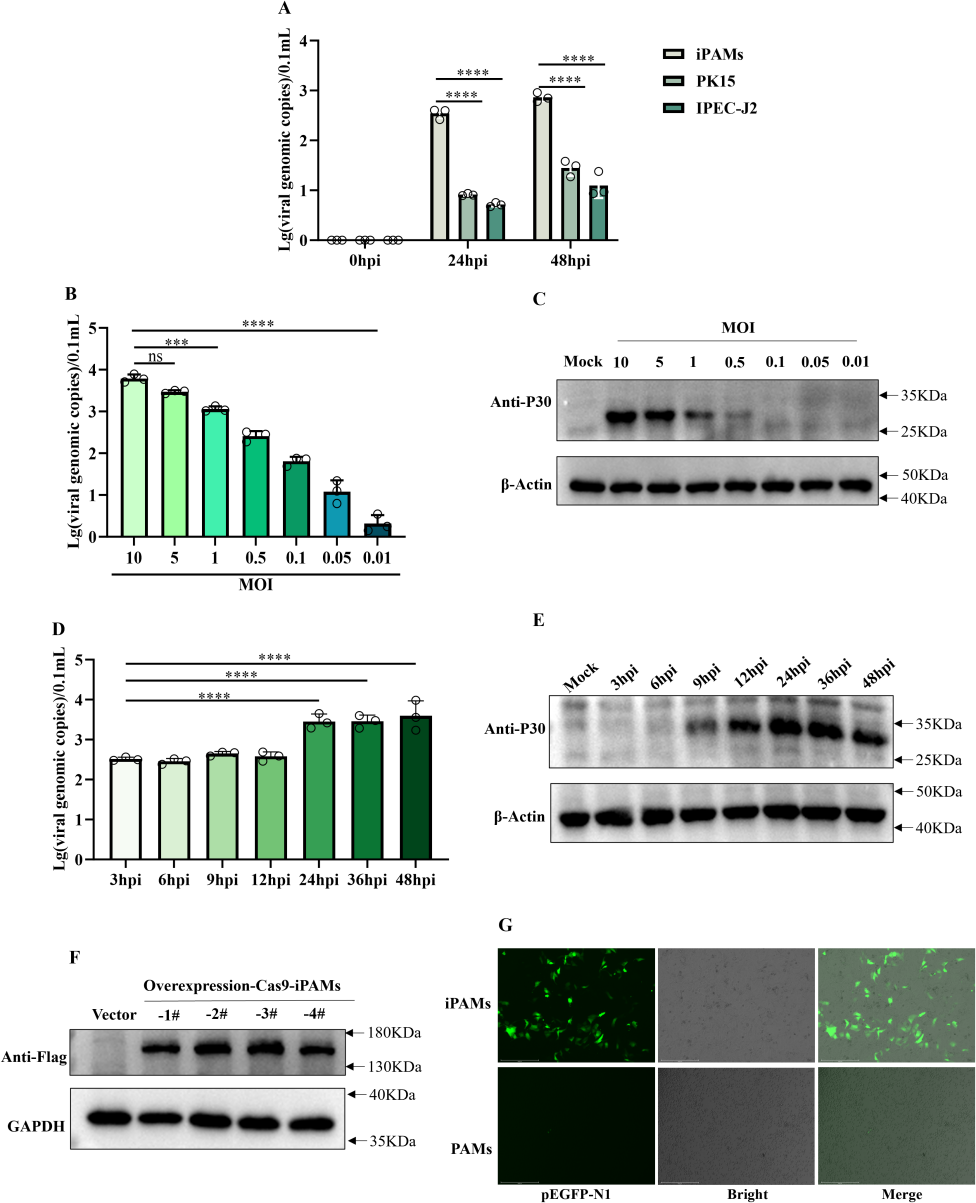
iPAMs are a relatively susceptible for SY-1 and have gene editing capabilities porcine macrophage cell line. **(A)** iPAMs, PK15, IPEC-J2 were infected with ASFV at MOI = 0.5. Viral genomic copies in supernatants were quantified using qPCR at 0, 24, and 48 h post-infection (hpi). **(B, C)** iPMAs were infected with ASFV at MOIs ranging from 0.01 to 10. Supernatants harvested and P30 protein at 24 hpi were analyzed for viral genome load using qPCR and western blotting, respectively. **(D, E)** iPAMs were infected with ASFV at MOI = 5, and supernatants and cell lysate were collected at 3, 6, 9, 12, 24, 36, 48, and 72 hpi to detect viral replication dynamics by qPCR and western blotting, respectively. **(F)** Western blotting analysis confirmed Cas9 protein expression in four clonal cell lines using an anti-Flag primary antibody and HRP-conjugated anti-rabbit secondary antibody. **(G)** iPAMs and PAMs transfected with the pEGFP-N1 plasmid were evaluated for green fluorescence protein expression 24 h post-transfection via fluorescence microscopy. The data are presented as the means ± SD by one-way analysis (***p < 0.001; ****p < 0.0001).

In order to observe the infectivity of the SY-1 strain to the passaged cell lines, we selected three porcine-derived passaged cell lines (PK15, IPEC-J2, iPAMs) to infect the SY-1 strain at MOI = 0.5 for 24h and 48h. qPCR showed that viral genome copy numbers were significantly higher in the iPAMs than in the other cell lines (**Figure 1A**). Thus, the iPAMs were choice as the candidate for further studies. Specifically, to characterize SY-1 infection of iPAMs in more depth, cells were infected with SY-1 at different MOIs, ranging from 0.01 to 10. Viral genome copies and western blotting showed that infection decreased with decreasing MOI (**Figures 1B, C**). To determine the replication kinetics of SY-1 in iPAMs, cells were infected at MOI = 5, and supernatants and cells were analyzed by qPCR and western blotting from 3–48 hpi, respectively. The viral load of supernatant increased by 24 hpi and stabilized at 36hpi and 48hpi, western blotting showed that the viral protein P30 of cells was detected at 9 h and increased with time by 24 hpi and remained unchanged at 36 hpi and 48 hpi (**Figures 1D, E**). To assess the suitability of iPAMs for functional genomics, we generated Cas9-overexpressing iPAMs monoclonal lines using lentiviral transduction. Western blotting analysis confirmed Cas9 protein expression in four independent clonal lines compared with that in the control cells (**Figure 1F**). Additionally, transfection efficiency was evaluated using the pEGFP-N1 plasmid. Fluorescence microscopy at 24 hpi showed higher green fluorescence in iPAMs than those observed in PAMs (**Figure 1G**).

These results confirm that although iPAMs supported higher initial SY-1 replication compared to other cell lines, but this replication plateaued early, indicating that SY-1 replicates only to a limited extent in iPAMs. However, as macrophages possessing gene-editing capability, they provide a valuable foundation for investigating virus-host interactions.

### Differences in ASFV infection between iPAMs and PAMs

**Figure 2:**
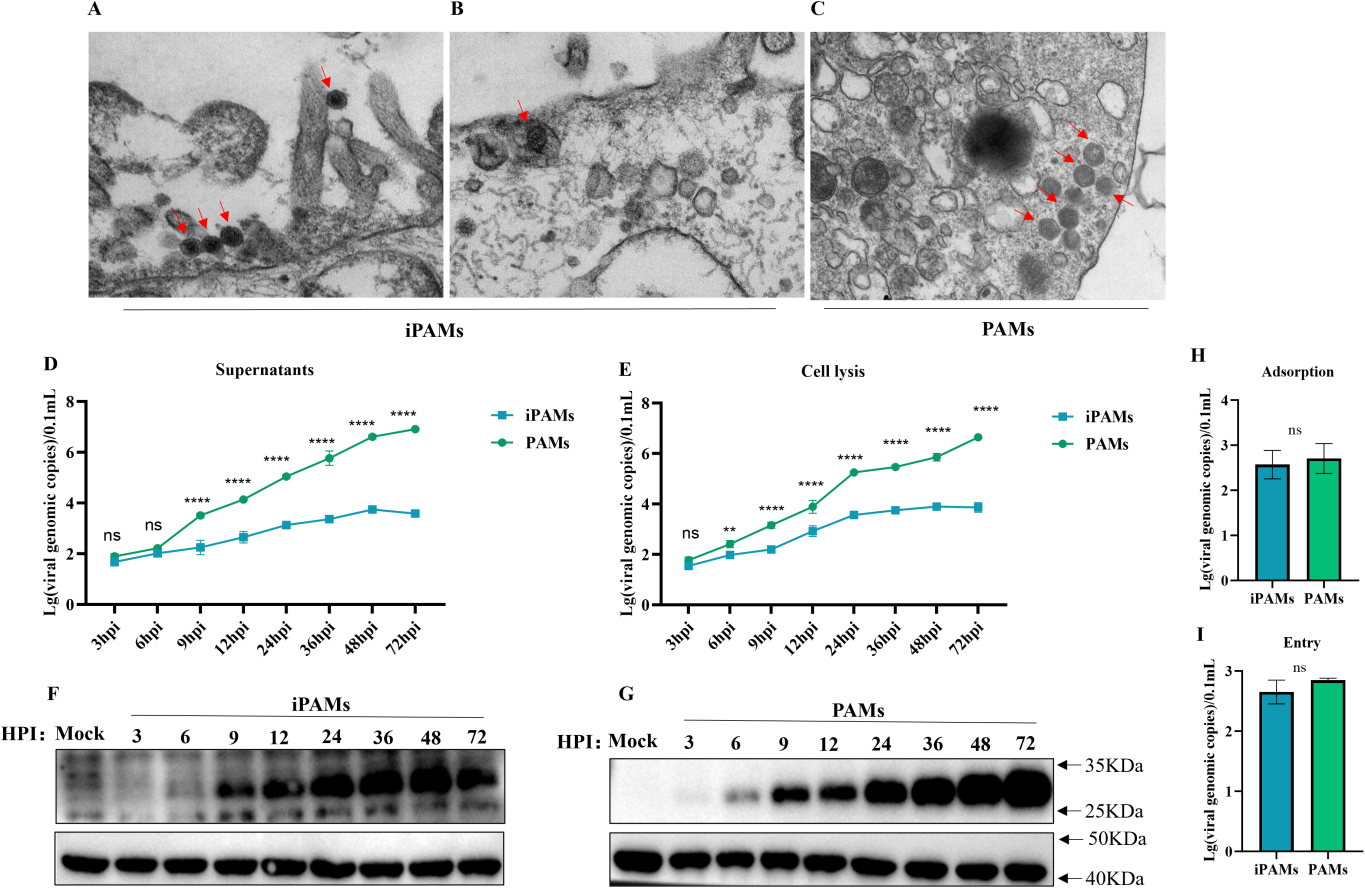
Differences in ASFV infection between iPAMs and PAMs. **(A-C)** Viral morphology of SY-1 infection with MOI = 5 for 24hpi in iPAMs and PAMs by transmission electron microscopy (TEM). Scale bars: 200nm. iPAMs and PAMs infected ASFV at MOI = 5 were sampled at 3–72 hpi **(D-G)**. Viral genomic copies in the supernatants **(D)** and cell lysates **(E)** were quantified via qPCR, and P30 protein in cell lysates was detected by western blotting from 3hpi to 72hpi in iPAMs **(F)** and PAMs **(G)**, respectively. Cells were incubated with ASFV (MOI = 5) at 4 °C for 1 h, washed thrice with cold PBS to remove unbound virus, and harvested for qPCR analysis of surface-bound viral genomes **(H)**. Following adsorption, cells were incubated at 37°C for 30 min in RPMI-1640 medium (5% FBS), washed thrice with PBS to remove the non-internalized virus, and lysed for quantification of the internalized viral genomes **(I)**. The data are presented as the means ± SD by one-way analysis (**p < 0.01; ****p < 0.0001).

To systematically compare the replication dynamics of the SY-1 strain in iPAMs and PAMs, we first observed viral morphology in iPAMs and PAMs using transmission electron microscopy (TEM). Typical ASFV particles with hexagonal capsids were observed in both cell types. In iPAMs cells, virions predominantly localized to the cell surface and pseudopodia (**Figure 2A**), and little was observed inside the cells (**Figure 2B**). In PAMs, viral particles were primarily detected within intracellular compartments (**Figure 2C**).

Next, we analyzed viral replication kinetics at multiple stages post-infection. iPAMs and PAMs were infected SY-1 at an MOI = 5, the viral loads of intracellular fractions and supernatants were detected by qPCR from 3 to 72 hpi, respectively and western blot was used to test the viral protein level of P30 in cells from 3 to 72 hpi. These results showed that both iPAMs and PAM cells were capable of being infected with the SY-1 strain, but iPAMs exhibited significantly lower viral genome copy numbers in both supernatants and intracellular compartments compared to PAMs (**Figures 2D, E**). In iPAMs, P30 protein levels remained stable from 24 to 48 hpi but exhibited decline by 72 hpi (**Figure 2F**). Conversely, PAMs the content of P30 protein increased significantly with time (**Figure 2G**). Meanwhile we could find that there are no significant differences in intracellular viral content were observed at 3 hpi. However, by 6 hpi, iPAMs displayed markedly lower intracellular viral loads compared to PAMs (**Figure 2E**). The supernatant viral loads showed delayed divergence, becoming statistically distinct only at 12 hpi (**Figure 2D**). The P30 protein did not appear until 9h in iPAMs, but was detected at 6h in PAMs (**Figures 2F, G**).

Finally, to assess whether this replication deficit stemmed from adsorption or invasion, we quantified viral adsorption (4 °C, 1 h) and entry (37 °C, 30 min post-adsorption). qPCR analysis revealed no significant differences in the number of bound or internalized viral genomes between iPAMs and PAMs (**Figures 2H**, **I**).

### Transcription divergence between iPAMs and PAMs during early ASFV infection

**Figure 3:**
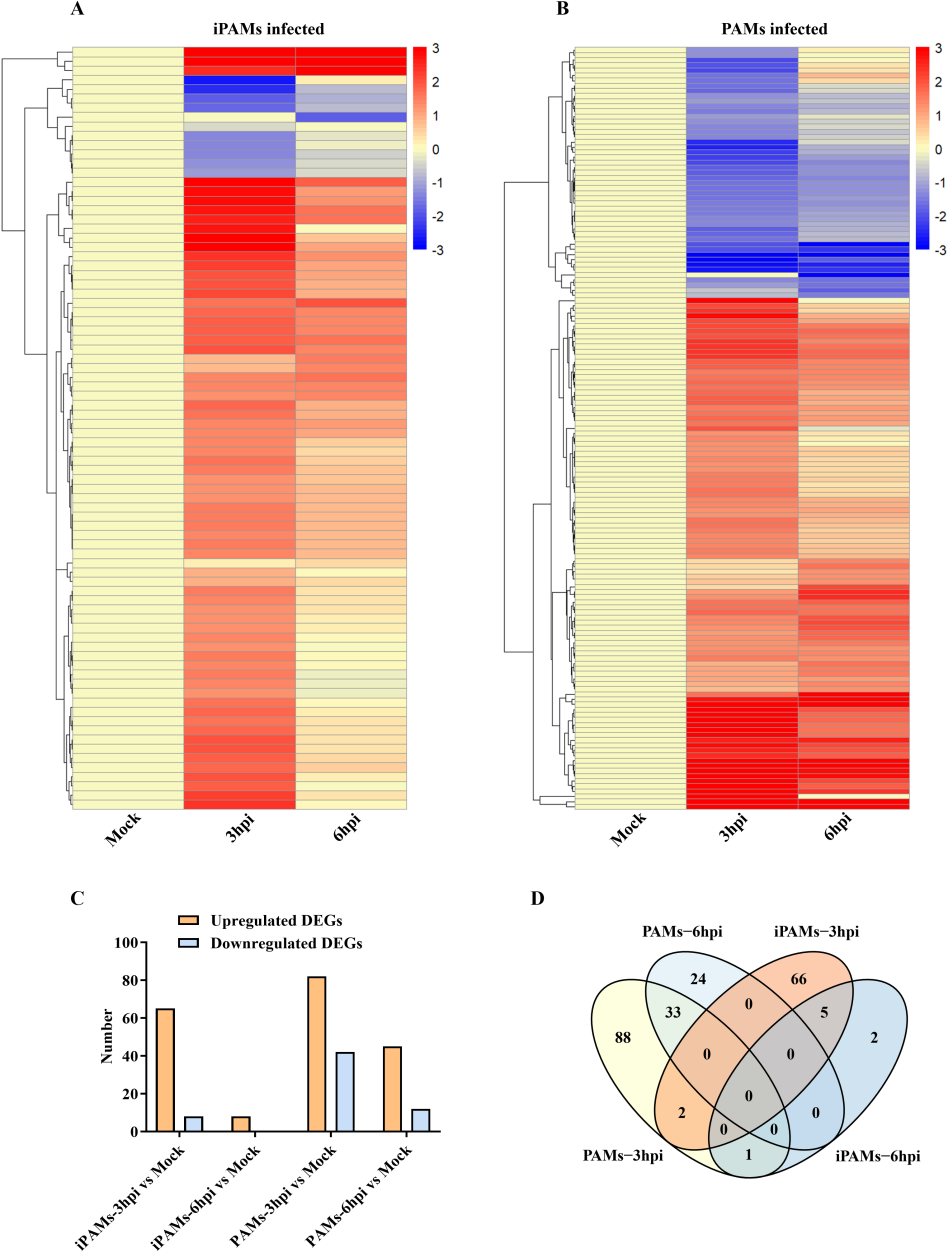
Transcription divergence between iPAMs and PAMs during early ASFV infection. **(A, B)** Heatmap analysis of differentially expressed genes (DEGs) between iPAMs and PAMs at 3 and 6 hpi. Rows represent genes; columns represent biological replicates. Color intensity reflects normalized expression levels (red: upregulation; blue: downregulation). **(C)** Bar graphs quantify upregulated (orange) and downregulated (pale blue) DEGs in iPAMs and PAMs at 3 and 6 hpi. **(D)** Venn diagram of DEGs in ASFV-infected PAMs and iPAMs.

We performed RNA-seq analysis of SY-1-infected iPAMs and PAMs (MOI = 5) at 3 and 6 hpi to investigate possible differences. Differentially expressed genes (DEGs) were defined as those with p < 0.05 and |log_6_ (fold change) | > 1, as detailed in **Supplementary Tables 3**–**6**. A heatmap of the DEGs showed distinct expression patterns between iPAMs and PAMs at 3 and 6 hpi (**Figure 3A**, **Figure 3B**). PAMs exhibited broad transcriptional remodeling (>120 DEGs at 3 hpi), whereas iPAMs showed a more restrained response (73 DEGs at 3 hpi), which is consistent with their attenuated viral replication phenotype. According to bar graph analysis results, iPAMs showed upregulation of 65 and 8 genes and downregulation of 8 and 0 genes at 3 h and 6 h, respectively, compared with that at 0 h. In contrast, PAM cells showed upregulation of 82 and 45 genes and downregulation of 42 and 12 genes at 3 and 6 hpi, respectively (**Figure 3C**). Venn diagram analysis highlighted that only three genes overlapped between 3 and 6 hpi in iPAMs, whereas 26 genes were common at both time points in PAMs. However, no common DEGs were detected between the iPAMs and PAMs at matched time points (**Figure 3D**).

### Functional enrichment analysis of transcriptomic responses to ASFV infection in iPAMs and PAMs

To explore the functional divergence between iPAMs and PAMs during early ASFV infection by performing GO and KEGG pathway analyses of the DEGs identified at 3 and 6 hpi in both cell lines.

**Figure 4:**
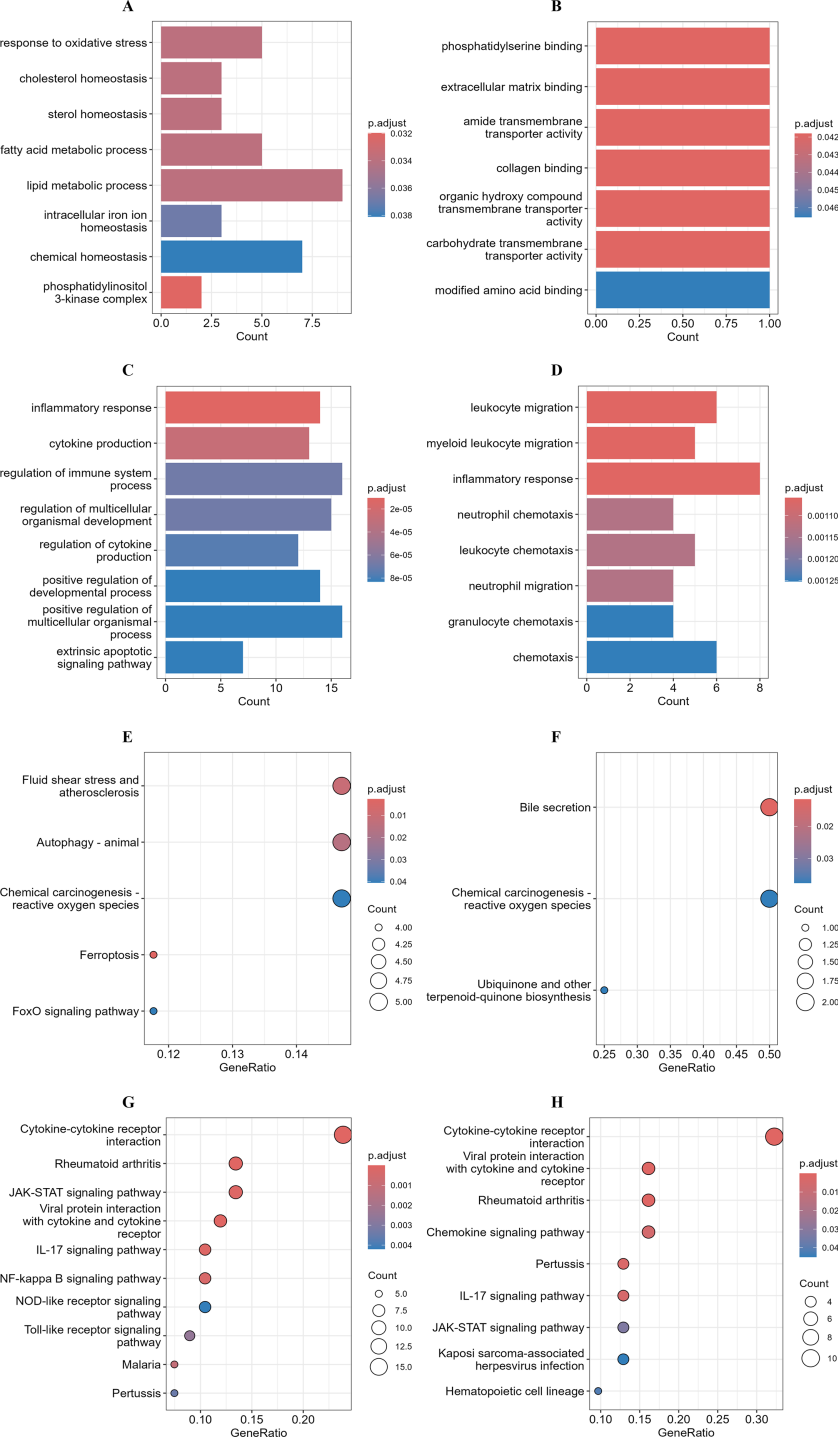
Functional enrichment analysis of transcriptomic responses to ASFV infection in iPAMs and PAMs. Gene Ontology (GO) enrichment and Kyoto Encyclopedia of Genes and Genomes (KEGG) pathway analyses of common DEGs identified through transcriptomic analysis between the ASFV-infected iPAMs and PAMs. **(A–D)** GO enrichment analysis of common up- and downregulated DEGs in of iPAMs at 3 hpi **(A)** and 6 hpi **(B)** and in PAM cells at 3 hpi **(C)** and 6 hpi **(D)**. **(E–H)** KEGG pathway analysis of common up- and downregulated DEGs in iPAMs at 3 hpi **(E)** and 6 hpi **(F)** and PAMs at 3 hpi **(J)** and 6 hpi **(H)**.

GO analyses of DEGs in the iPAMs showed enrichment in processes related to lipid metabolism (such as fatty acid metabolic process and lipid metabolic process), chemical homeostasis, and oxidative stress response at 3 hpi (**Figure 4A**) and molecular functions including phosphatidylserine binding, extracellular matrix binding, and transmembrane transporter activity (such as amide, carbohydrate, and organic hydroxy compounds) at 6 hpi (**Figure 4B**). These results suggest alterations in membrane dynamics and nutrient transport. In contrast, GO analyses of the PAM DEGs showed strong associations with inflammatory responses (such as cytokine production and immune system regulation) and regulation of multicellular organismal development at 3 hpi (**Figure 4C**) and shift in GO-enriched terms toward leukocyte migration (such as myeloid leukocyte migration and chemotaxis) and sustained inflammatory signaling at 6 hpi (**Figure 4D**).

KEGG pathway analysis of iPAMs showed enrichment in autophagy (autophagy-animal), oxidative stress (chemical carcinogenesis-reactive oxygen species), and vascular pathophysiology (fluid shear stress and atherosclerosis) at 3 hpi (**Figure 4E**) and pathways related to bile secretion and persistent oxidative stress-related carcinogenesis at 6 hpi (**Figure 4F**). KEGG analysis of the DEGs in the PAMs highlighted cytokine–cytokine receptor interactions and viral protein–cytokine interplay (**Figure 4G**). The continued enrichment of cytokine signaling and immune cell recruitment pathways at 6 hpi highlighted sustained inflammatory engagement (**Figure 4H**).

### Effect of siRNA-mediated gene knockdown on ASFV infection in iPAMs

**Figure 5:**
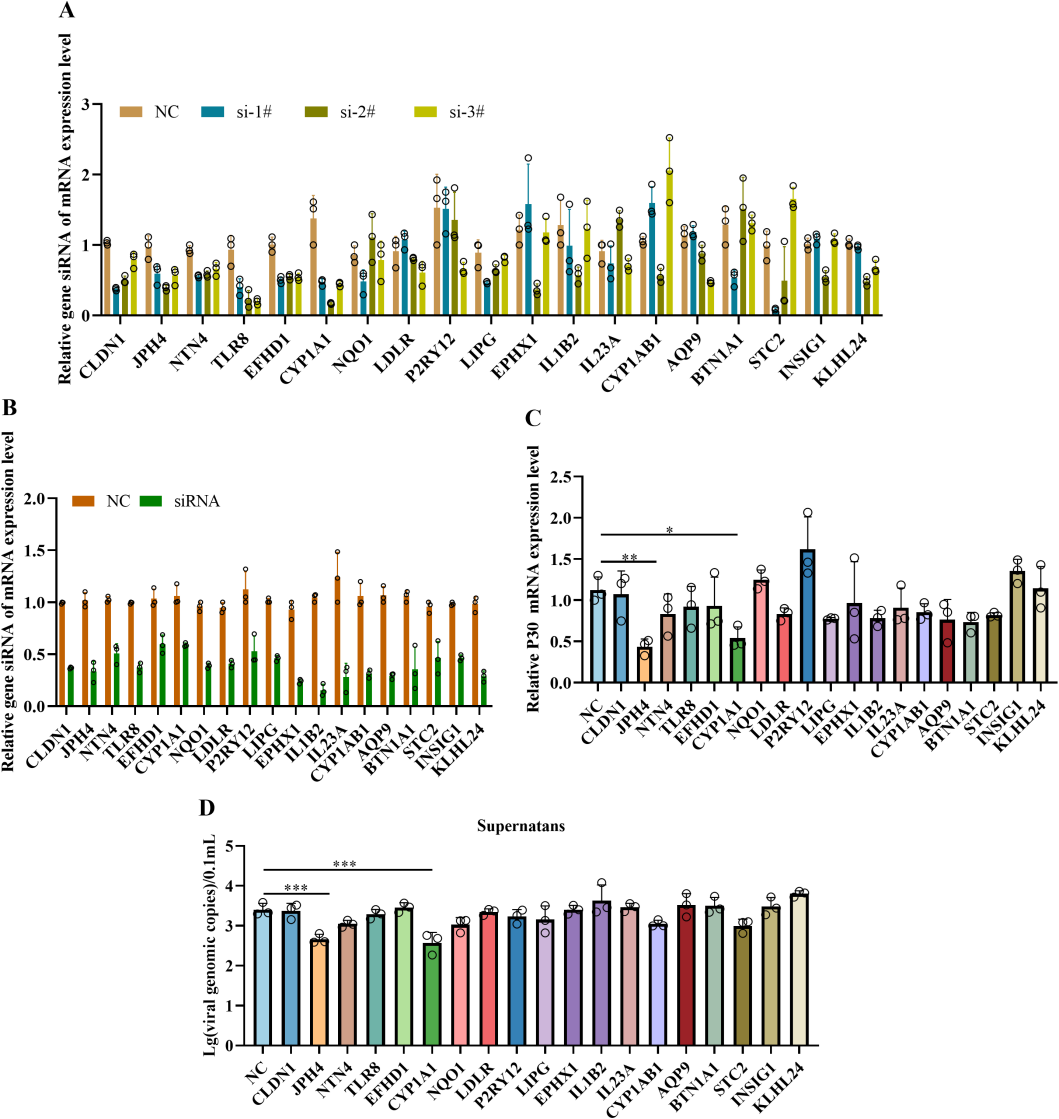
Effect of siRNA-mediated gene knockdown on ASFV infection in iPAMs. iPAMs were transfected with siRNA for 24 h to assess knockdown efficiency using RT-qPCR **(A)**. Following transfection, cells were infected with ASFV (MOI = 5) for an additional 24 hpi, and cellular RNA was analyzed using RT-qPCR to quantify residual siRNA levels **(B)** and viral P30 mRNA expression **(C)**, whereas supernatants were subjected to qPCR to determine viral genomic copy numbers **(D)**. This experiment was repeated thrice. The data are presented as the means ± SD by one-way analysis (*p < 0.5; **p < 0.01; ***p < 0.001).

Considering the differences in transcript levels between the iPAMs and PAMs, we prioritized the top three candidate genes based on transcriptomic analysis (|log_6_ FC| > 1.0 in the iPAMs and PAMs at 3 and 6 hpi) to explore the effects on viral replication. As PAMs exhibit low transfection efficiency, validation was performed using the iPAMs. Three siRNAs were designed to validate the knockdown effect of each gene, and the RT-qPCR results showed that at least one siRNA per gene was successfully knocked-down (**Figure 5A**). Next, we selected at least one siRNA per gene that exhibited ≥50% mRNA knockdown efficiency to further validate the impact on viral infection (**Figure 5B**). RT-qPCR analysis showed that the knockdown of *JPH4* and *CYP1A1* significantly reduced P30 mRNA levels compared with that in the control (**Figure 5C**). Additionally, qPCR analysis of viral genome copies in supernatants harvested at 24 hpi showed a significantly decrease in viral replication after *JPH4* and *CYP1A1* knockdown compared with that observed for the control and other genes (**Figure 5D**).

### Effect of ectopic expression of genes on ASFV infection

**Figure 6:**
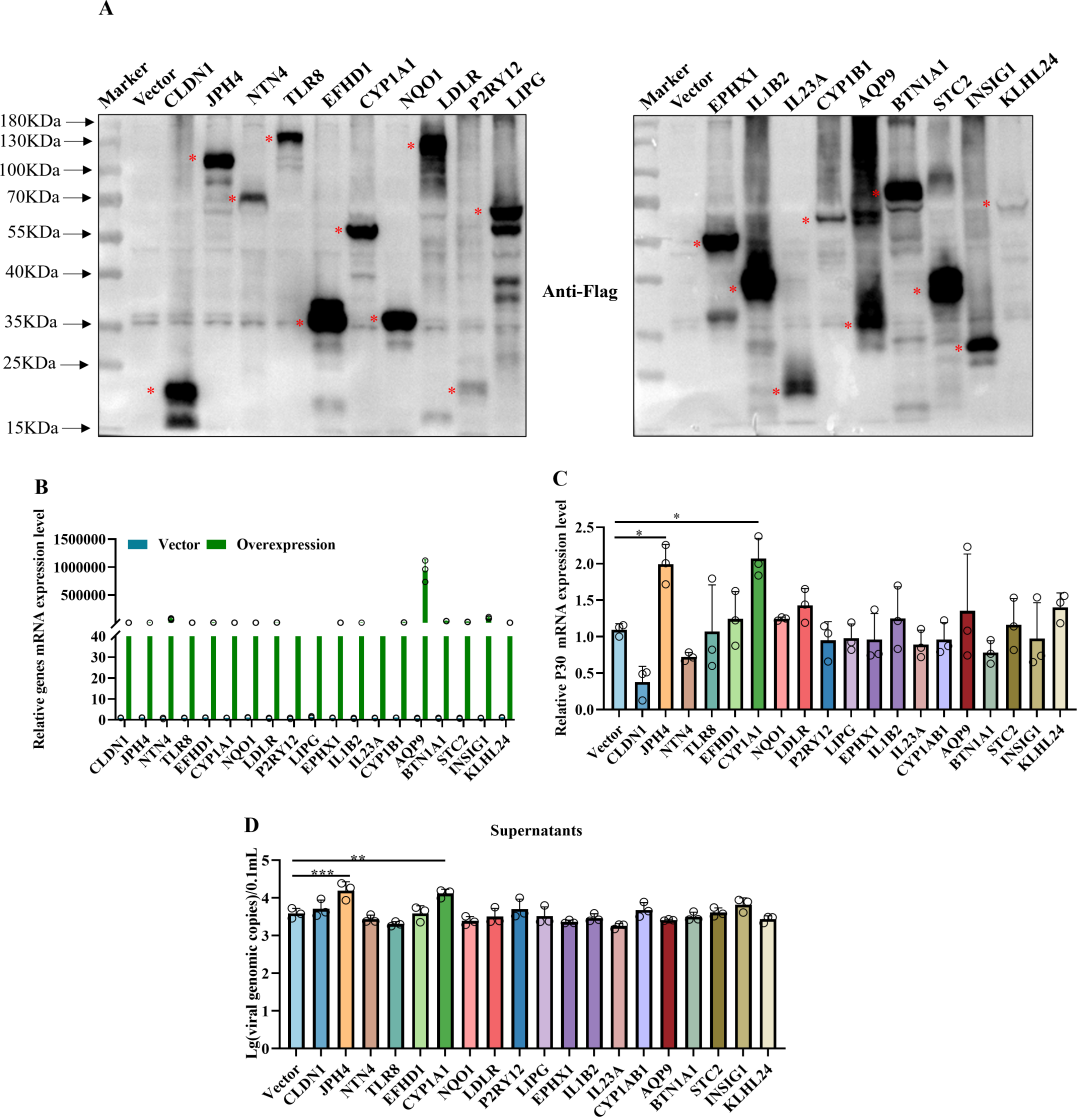
Effect of ectopic expression of genes on ASFV infection. **(A)** iPAMs were transfected with genes of Flag-tagged plasmids for 24 h, and western blotting was performed to test gene expression. **(B-D)** iPAMs were transfected with overexpression genes for 24 h and infected with ASFV at MOI = 5 for 24 h; cells were collected to detect the mRNA levels of genes **(B)** and P30 **(C)** via RT-qPCR. The supernatants were collected to detect the viral genomic copies using qPCR **(D)**. The data are presented as the means ± SD by one-way analysis (*p < 0.5; **p < 0.01; ***p < 0.001).

Western blotting analysis confirmed the successful expression of the transfected genes, which validated the efficient expression of the Flag-tagged constructs in the iPAMs (**Figure 6A**). Furthermore, RT-qPCR quantified the transfection efficiency and overexpression levels by measuring the mRNA levels of the target genes (**Figure 6B**). We assessed the mRNA levels of the viral structural protein P30 normalized to that of GAPDH (**Figure 6C**). Concurrent analysis of viral copy numbers in cell culture supernatants using qPCR provided a complementary measure of viral progeny production (**Figure 6D**). These findings indicated that *JPH4* and *CYP1A1* overexpression promotes ASFV replication in iPAMs.

## Discussion

Various cell lines, such as African green monkey kidney epithelial cells (MA-104), immortalized porcine kidney-derived macrophages (IPKMs) (22, 23), Verda Reno (Vero), monkey kidney tissue-derived cells (COS-1) and human embryonic kidney (HEK293T) (24–26), exhibit inefficient viral replication compared to primary cells. However, after multiple passages, the virus is prone to genetic variation, resulting in changes in virulence and immunogenicity, potentially leading to inaccurate results in the construction of viral mutant or deletion strains. For example, the deletion of EP296R does not affect the replication or virulence of the virus in pigs, a finding that contrasts with the results obtained using the BA71V strain (27–29). One of the probable reasons is that the genomic differences of the BA71V strain during its adaptation to Vero cells led to the deletion of 11 genes belonging to the MGF360/505 family (30, 31). Deletion of the E165R gene in a natural, highly virulent field isolate did not significantly affect ASFV replication in macrophages, which is significantly different from the results of previous studies (32). The same reason may be that the previous viral strains were adapted to grow in Vero cells (11). Therefore, to facilitate the in-depth study of ASFV, it is crucial to find cell lines that do not require the adaptive passage of the virus.

In our study, transcriptomic analysis was performed at 3 and 6 hpi based on the viral replication cycle and detection results of viral loading and P30 protein between iPAMs and PAMs. GO and KEGG analyses showed there was rapid activation of pro-inflammatory pathways, characterized by a robust upregulation of cytokines (e.g., *IL1B*) and chemokines (e.g., *CXCL*) in PAMs. This is consistent with the cytokine storm dynamics reported by Zheng et al. (33, 34) in primary macrophage infections. In contrast, iPAMs exhibited minimal inflammatory activation but instead mobilized oxidative stress mitigation programs, including oxidative stress and antioxidant response (*CYP1A1*, *CYP1B1*, and *HMOX1*), autophagy (*ULK1*, *WIPI1*, and *SQSTM1/p62*), and lipid metabolism and cholesterol biosynthesis (*INSIG1*). These findings suggested that the host response in iPAMs were distinct from those of the PAMs during ASFV infection. Additionally, we preliminary verified that JPH4 and CYP1A1 could influence virus replication through overexpression and knockdown experiments in iPAMs (**Figures 5**, **6**). We also analyzed the differential expression of JPH4 and CYP1A1 during the early stages of infection in iPMAs and PAMs. Results showed that CYP1A1 was markedly upregulated in iPAMs (**Supplementary Figure 1A**), and JPH4 was significantly downregulated in PAMs (**Supplementary Figure 1B**). This finding provides further evidence that these two genes may regulate viral replication and further demonstrates the feasibility of conducting early-stage transcriptomic analyses. Concurrently, we conducted JPH4 and CYP1A1 knockdown experiments in PAMs, yielding results consistent with those in iPAMs (**Supplementary Figure 2A-I**). This indicated that JPH4 and CYP1A1 were key factors influencing viral replication. Furthermore, we observed that following knockdown of JPH4 and CYP1A1 in PAMs, mRNA levels of TNF-α remained unchanged, but JPH4 upregulated mRNA expression of IFN-β, whereas CYP1A1 showed no change (**Supplementary Figure 2J, K**). We hypothesis that JPH4 may exert its effects through interferon regulation, whereas CYP1A1 influences viral replication via other ways in PAMs.

Our current research has several limitations. On one hand, we only conducted preliminary investigations into the roles of JPH4 and CYP1A1 in IPAM cells, with limited validation in more susceptible PAM cells and no in-depth exploration of the underlying mechanisms. One the other hand, our gene selection focused solely on significantly differentially expressed candidate genes in the transcriptome, without functional validation of pro-viral factors specifically highly expressed in PAMs. Such factors may be key to explaining why PAMs more strongly support viral replication. Therefore, in subsequent studies, we plan to further explore the mechanisms underlying JPH4 and CYP1A1 and we will focus on genes uniquely highly expressed in PAMs within the transcriptome data, conducting systematic functional screening to fill current gaps.

Overall, while this study has not fully resolved the core scientific questions, the transcriptomic analysis and preliminarily validated host factors could provide a direction for subsequent research and hold exploratory value.

## Data Availability

The sequencing data generated in this study have been deposited in the NCBI Sequence Read Archive (SRA) under the accession number PRJNA1240594.

## Supplementary material

Supplementary Table 1 The siRNA primers used during designing.

Supplementary Table 2 Primers used for qRT-PCR.

Supplementary Table 3 DEGs of porcine alveolar macrophage (PAM) cells at 3 h post-infection (hpi) compared with that of the mock.

Supplementary Table 4 DEGs of PAM cells at 6 hpi compared with that of the mock.

Supplementary Table 5 DEGs of iPAMs of in 3 hpi compared with that of the mock.

Supplementary Table 6 DEGs of iPAMs at 6 hpi compared with that of the mock.

Supplementary Figure 1 Early infection expression of JPH4 and CYP1A1 in the two cell types. **(A, B)** iPAMs and PAMs infected with ASFV at MOI = 1 for 3 hpi and 6 hpi. The cells were collected to detect the mRNA levels of genes and P30 in iPAMs and PAMs, respectively.

Supplementary Figure 2 Knockdown of JPH4 and CYP1A1 in PAMs. **(A-D)** PAMs were transfected with JPH4 and CYP1A1 for 24 h to assess knockdown efficiency using RT-qPCR **(A, C)** and western blot **(B, D)**, respectively. Following transfection, cells were infected with ASFV (MOI = 0.1) for an additional 24 hpi, and cellular RNA was analyzed using RT-qPCR and western blot to quantify residual siRNA levels **(E, G)**, mRNA expression of P30, IFN-β and TNF-α **(I-K)** and protein level of P30, JPH4 and CYP1A1**(F, H)**. This experiment was repeated thrice. The data are presented as the means ± SD by Two-way analysis (**p* < 0.5; ***p* < 0.01).
