# Supplementary figures and images for: Transcriptome analysis of differences in the infection of African swine fever virus (SY-1 strain) in iPAMs and PAMs
Source: Front Immunol. 2025 Dec 5;16:1692373. doi: 10.3389/fimmu.2025.1692373 (PMC12714971; doi:10.3389/fimmu.2025.1692373)

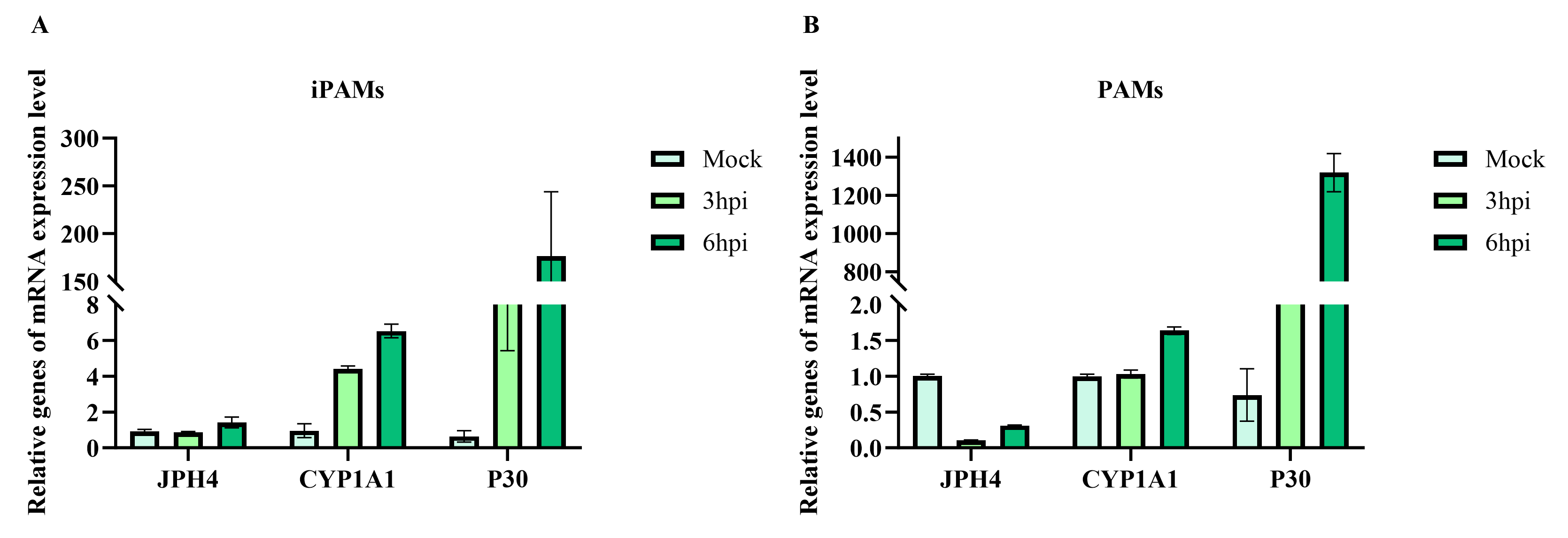

Supplement: Supplementary Figure 1 — Early infection expression of JPH4 and CYP1A1 in the two cell types. (A, B) iPAMs and PAMs infected with ASFV at MOI = 1 for 3 hpi and 6 hpi. The cells were collected to detect the mRNA levels of genes and P30 in iPAMs and PAMs, respectively. [file Image1.tif]

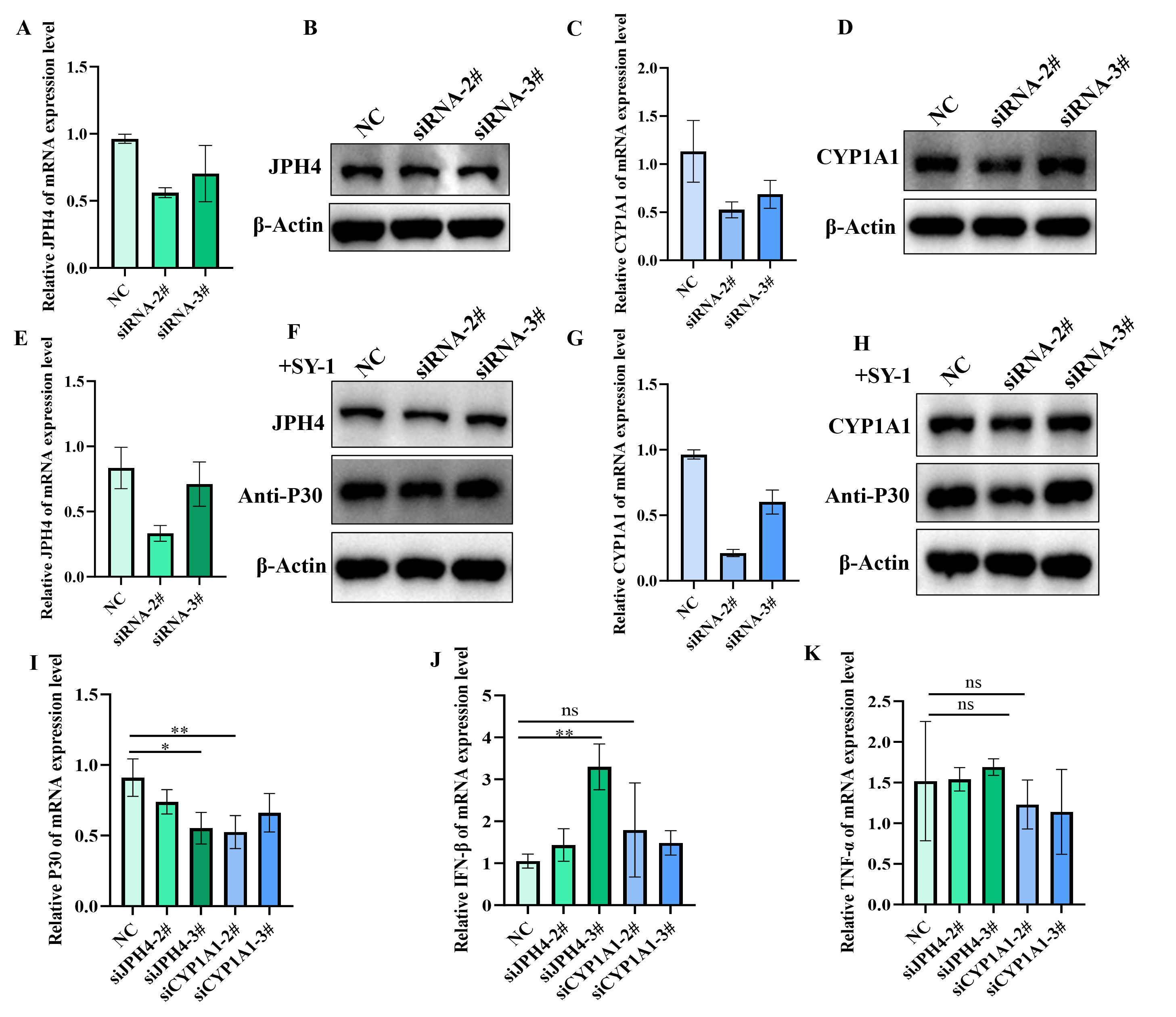

Supplement: Supplementary Figure 2 — Knockdown of JPH4 and CYP1A1 in PAMs. (A-D) PAMs were transfected with JPH4 and CYP1A1 for 24 h to assess knockdown efficiency using RT-qPCR (A, C) and western blot (B, D), respectively. Following transfection, cells were infected with ASFV (MOI = 0.1) for an additional 24 hpi, and cellular RNA was analyzed using RT-qPCR and western blot to quantify residual siRNA levels (E, G), mRNA expression of P30, IFN-β and TNF-α (I-K) and protein level of P30, JPH4 and CYP1A1(F, H). This experiment was repeated thrice. The data are presented as the means ± SD by Two-way analysis (*p < 0.5; **p < 0.01). [file Image2.tif]
